# Reimbursement Potential of Collaborative Care Model (CoCM) Billing Codes for Opioid Use Disorder Co-Occurring with Mental Disorders: Descriptive Estimates from a Pragmatic Trial

**DOI:** 10.1007/s10488-026-01503-z

**Published:** 2026-04-27

**Authors:** Alex R. Dopp, Rebecca L. Weir, Grace M. Hindmarch, Lia Pak, Sapna Mendon-Plasek, Michael Schoenbaum, Jasen Christensen, Valerie Carrejo, Caroline Bonham, Miriam Komaromy, Katherine E. Watkins

**Affiliations:** 1 RAND, Santa Monica, CA, USA; 2 Fielding School of Public Health, Department of Health Policy and Management, University of California, Los Angeles, Los Angeles, CA, USA; 3 Division of Services and Intervention Research, National Institute of Mental Health, Bethesda, MD, USA; 4 School of Medicine, Department of Psychiatry and Behavioral Sciences, University of New Mexico, Albuquerque, NM, USA; 5 School of Medicine, Department of Family and Community Medicine, University of New Mexico, Albuquerque, NM, USA; 6 Grayken Center for Addiction, Boston Medical Center, Boston, MA, USA; 7 Chobanian and Avedisian School of Medicine, Division of General Internal Medicine, Boston University, Boston, MA, USA

**Keywords:** Collaborative care, Behavioral health integration, Evidence-based practice, Health care financing, Billing codes, Sustainability

## Abstract

In 2017, the U.S. Centers for Medicare and Medicaid Services approved reimbursement for billing codes specific to the Collaborative Care Model (CoCM), an evidence-based practice for improving access and quality of behavioral health services in primary care. However, it remains unclear how reimbursement through these billing codes aligns with applications of CoCM for complex patient populations, such as those with co-occurring mental and substance use disorders. We examined the reimbursement potential of CoCM intervention activities documented during a pragmatic clinical trial of CoCM for patients with opioid use disorder co-occurring with depression and/or post-traumatic stress disorder. We defined reimbursement potential based on federal (i.e., Medicare) CoCM billing code rules and reimbursement rates, as of 2024. Across 381 patients and 10 care managers (i.e., the CoCM interventionists), we documented 90,996 total intervention activity minutes in the project’s care management registry. Under ideal conditions where all CoCM billing codes can be and are used, a maximum of 56% of minutes would be billable, generating $91.61 reimbursement per hour of care management. Reimbursement potential was lower under restrictive billing conditions and settings, resulting in $79.32 per hour. Most commonly, minutes were unbillable due to not meeting CoCM service requirements or exceeding maximum billable time for a month. Sensitivity analyses showed that reimbursement potential could be notably lower in alternative scenarios (e.g., $39.63–$43.04 per hour with minimum plausible Medicaid billing rates). Continued attention is needed to align CoCM reimbursement potential with clinical needs to ensure feasibility and sustainability with complex patient populations.

**Supplementary Information:**

The online version contains supplementary material available at 10.1007/s10488-026-01503-z.

Approximately 21.5 million U.S. adults (8.4% of the adult population) had co-occurring mental and substance use disorders in the past year (Substance Abuse Mental Health Services Administration [SAMHSA], 2023). Compared to mental or substance use disorders alone, co-occurring disorders are associated with higher treatment costs (Hoff & Rosenheck, 1999), higher morbidity and mortality (Rees et al., 2022), and worse treatment outcomes (Burns et al., 2005). Unfortunately, many individuals with co-occurring mental and substance use disorders never receive treatment, and even among those who do, most do not receive treatment for both conditions (Han et al., 2017; Jones & McCance-Katz, 2019; Watkins et al., 2001). Primary care is an important setting to treat co-occurring behavioral health problems because, compared to specialty care, (1) visits are more frequent and therefore patients are more likely to be identified and able to access needed care (Murray-Krezan et al., 2023; SAMHSA, 2020); and (2) patients report experiencing less stigma (Incze et al., 2023). Furthermore, evidence-based models exist for integrated primary care treatment of mental and substance use disorders (National Academies of Sciences & Medicine, 2021; Santos et al., 2024). The current study examined how available financing for one such integrated care model, the collaborative care model (CoCM), aligns with its use in treating co-occurring mental and substance use disorders.

CoCM is an evidence-based model of care shown to improve healthcare access and outcomes for individuals with behavioral health disorders in primary care (Reist et al., 2022; Unützer et al., 2002). CoCM consists of a team of providers who deliver evidence-based treatments; beyond the usual primary care providers (i.e., physician, nurse practitioner, or physician assistant) and behavioral health providers, CoCM specifically includes (a) a care manager who coordinates care between the patient and members of the care team and monitors treatment adherence, tolerance, and response; and (b) a psychiatric consultant who provides psychiatrist-level expertise to the team (AIMS Center, 2023a). The model uses patient-centered principles to help patients meet their treatment goals and to maximize engagement of the eligible patient population. In addition to strong evidence demonstrating improvements in depression, anxiety, and alcohol use disorders (Archer et al., 2012; Unützer et al., 2002; Woltmann et al., 2012), CoCM is cost-effective and can improve quality of care while simultaneously reducing overall healthcare costs (Unützer et al., 2008). However, the effectiveness of CoCM for patients with co-occurring mental and substance use disorders remains understudied, which is concerning because engaging and treating those patients is more complex and challenging (McGovern et al., 2006; Snell-Rood et al., 2021). Currently, a series of clinical trials funded by the U.S. National Institute of Mental Health (2019) provide an opportunity to study the effectiveness and implementation of CoCM for opioid use disorder co-occurring with mental disorders, including consideration of the scalability of tested models through available billing codes.

The Centers for Medicare and Medicaid Services (CMS) approved reimbursement for Medicare Behavioral Health Integration (BHI) codes, including separate payments for using CoCM, starting in 2017 (Centers for Medicare & Medicaid Services, 2023a). This advance was meant to encourage use of CoCM and other BHI practices in primary care clinics, offering dedicated insurance reimbursement for the first time (Press et al., 2017). However, approval of the CoCM billing codes only made them immediately available for Medicare beneficiaries. To date, many commercial payers have started to reimburse the codes (Carlo et al., 2019; Raney, 2020) as well as 38 state Medicaid programs (C. McNutt, personal communication, June 12, 2025). Uneven adoption of CoCM billing codes among payers has limited the reach of the model, yet the codes remain important to sustainably financing CoCM at scale (Carlo et al., 2018; Raney, 2020).

Use of CoCM billing codes has increased over time, but significant challenges contribute to continued variation in use of the codes and gaps in access to CoCM (Davenport et al., 2025). Notably, research has documented concerns about the adequacy of reimbursement when delivering CoCM to patients with mental disorders. In a qualitative study of healthcare organizations using the CoCM billing codes, a salient concern was lost revenue when requirements for the codes were not met (Carlo et al., 2019). Another study (Chang et al., 2023) examined the percentage of time spent on CoCM that was not eligible for reimbursement due to falling outside specified time requirements. There was considerable variability in the proportion of minutes below minimum (2–60%) or above maximum (1–13%) time requirements, and qualitative interviews with providers revealed related concerns about sustainability of supporting CoCM through these billing codes. Key barriers to delivering CoCM in ways that fully met billing requirements included limited workforce capacity for model-specific roles and challenges with patient engagement (Chang et al., 2023; Davenport et al., 2025). Given that the reimbursement provided by CoCM billing codes may be inadequate even when applied to care for single behavioral health disorders, it is particularly important to examine how such reimbursement aligns with using CoCM in treatment of patients with co-occurring mental and substance use disorders.

The current study applied CoCM billing codes to data from a pragmatic clinical trial testing CoCM for adults with opioid use disorder and co-occurring mental disorders. We sought to answer two interrelated research questions: (1) How much of CoCM activities with patients were eligible and ineligible for reimbursement through the existing billing codes? and (2) What is the reimbursement potential per hour of collaborative care? To answer the first question, we identified the proportion of minutes eligible and ineligible for reimbursement through CoCM and general BHI billing code rules, and we examined the reasons for ineligibility. For the second question, we assessed the reimbursement potential for CoCM by examining the expected revenue per hour of care manager time assuming standard reimbursement rates set by Medicare; investment of resources in the care manager role is an important added expense of CoCM. Across both research questions, we considered a variety of plausible scenarios that would affect reimbursement, including the billing codes available for reimbursement, type of primary care setting, and variation in key assumptions of our analytic model.

## Method

### Study Design and Setting

This study is a descriptive analysis of activities recorded during CoCM intervention delivery during CLARO (Collaboration Leading to Addiction Treatment and Recovery From Other Stresses), a pragmatic randomized controlled trial of CoCM for opioid use disorder and co-occurring mental disorders (Watkins et al., 2026). Data came from CoCM delivered in primary care clinics from two health systems in New Mexico (11 clinics total) and one health system in California (three clinics total). Eight clinics were Federally Qualified Health Centers (FQHCs; in the United States, FQHCs provide primary care to underserved patients and communities regardless of their ability to pay [Department of Health and Human Services, 2017]) and six clinics were from two non-FQHC systems (one academic medical center, one county health system). Two health systems (four clinics total) involved in the trial were excluded from the current analysis because they never successfully implemented CoCM (i.e., no or very limited patient enrollment, resulting in premature discontinuation as a trial site). Across the 14 included clinics, 10 community health workers filled the role of care managers who coordinated with psychiatric consultants, primary care teams, and therapists. We referred to our psychiatric consultants as behavioral health consultants (BHCs) because they were board-certified in addiction medicine or addiction psychiatry, which is not typically expected of CoCM consultants. Care managers documented activities in a standalone, customized patient registry that was standardized across clinics.

From January 2021 to June 2024, patient participants who enrolled in the CLARO trial at any clinic were randomized to either receive CoCM (i.e., assigned to a care manager) or enhanced usual care, which included availability of evidence-based treatments but no support from a care manager. To be eligible for enrollment, participants needed to be (a) adults of legal age who (b) screened positive for probable opioid use disorder, plus probable co-occurring depression and/or post-traumatic stress disorder, on validated screening tools (Adam et al., 2019; Kroenke et al., 2001; Prins et al., 2016); (c) considered the primary care clinic their usual source of care; (d) spoke and understood English or Spanish; and (e) did not require immediate medical or psychiatric intervention. Patients were identified through screening in clinic waiting rooms, referrals from clinic providers and staff, outreach based on documented diagnoses in clinic medical records, and self or peer/family referral through the study website. The CLARO trial had a final enrollment of 797 patients, of whom 381 were included in our study because they (f) were assigned to CoCM in a health system that successfully implemented the model and (g) had documented intervention activity (one patient assigned to CoCM was excluded due to not having any CoCM registry entries).

### CoCM Intervention

CoCM is defined by core principles (AIMS Center, 2023a), which we tailored for OUD and mental disorders and tested in the CLARO trial (Osilla et al., 2022; Watkins et al., 2026). Our approach to CoCM is grounded in a patient-centered care team that includes primary care providers offering evidence-based medication treatments for opioid use and mental disorders, behavioral health providers offering evidence-based psychotherapies, and the care manager and BHC coordinating a shared treatment plan. We also tailored an existing CoCM patient registry for our population; registries are caseload tracking tools that care managers use to document patient encounters and results from measurement-based care (MBC; i.e., routine measurement of clinical outcomes that guides treatment planning) for the population of patients assigned to CoCM. In CLARO, we offered CoCM for a standard 6-month intervention period; some patients received CoCM for longer based on clinical need, but all analyses focus on intervention activities that occurred in the 6-month period.

### Data Source

Data for this study was drawn from the CLARO CoCM registry. Care managers were trained to log details on each CoCM patient encounter – including time spent, activities conducted, notes on patient status, and MBC results – in the registry. Care managers also used the registry to note activities such as patient outreach attempts and consultations with the BHC. We used this documentation of care manager activities to estimate the amount of time spent on CoCM activities. However, it is important to note that the registry documentation was originally completed for clinical use only; all CoCM activities were funded by the trial and clinics did not bill for care manager services. We considered the potential impact of systematic differences between our documentation and registries used for billing purposes while building our analytic model and testing the impact of our assumptions (i.e., sensitivity analyses).

### Analysis

We used SAS 9.4 for data management and analyses. Given that we focused on analysis of documented activities, we did not impute data assumed to be missing (i.e., undocumented activities could not be billed). Our analysis included three main steps. First, we calculated the total number of care manager minutes documented. Then, we determined which of those minutes would have been eligible for reimbursement by applying decision rules modeled on current (as of 2024) CoCM and general BHI billing code requirements. Third, we calculated the expected reimbursement for the billable minutes using standard 2024 Medicare reimbursement rates for each code, and the average reimbursement rate per hour of care management. Given that payers vary in which codes they reimburse, we defined eligible minutes two ways: (1) an expansive scenario assuming all codes were billable and (2) a restrictive scenario with only the most frequently reimbursed codes. Within each scenario, we separately calculated eligible minutes and reimbursement rates for FQHCs and non-FQHCs, as they had different billing codes and rules. Finally, following the main analysis, we ran sensitivity analyses (detailed later) to test key assumptions of the model.

#### Total CoCM Minutes

We first compiled all registry data from the six-month period after patient randomization into CoCM, across all patients, to determine the total number of CoCM minutes that care managers documented throughout the study period. This involved summing all minutes spent on patient contact attempts, BHC consultations, and care manager encounters with the patient. In the registry, care managers could enter the number of minutes for encounters and BHC consultations. Contact attempts did not allow time entry, so we assumed each took five minutes based on input from the care managers and their supervisors.

#### Reimbursement Eligibility

We used available federal guidance to characterize care manager minutes as eligible or ineligible for reimbursement through CMS BHI billing codes for (a) CoCM and (b) other BHI services that have sufficient overlap in clinical activities and requirements that CoCM activities could be billed to them (Centers for Medicare & Medicaid Services, 2023a). Different billing codes are relevant depending on the timepoint (first month of CoCM vs. subsequent months), services provided, and whether the clinic is an FQHC. Table 1 lists all billing codes that we considered for reimbursement eligibility, including which codes were included in the restrictive scenario based on guidance about code availability (AIMS Center, 2023b, 2023d). We assumed that the two FQHC codes were billable in both scenarios because we found no indication that one code is ever billable without the other.

**Table 1:** Descriptions of billing codes used with the collaborative care model

Billing Code	Description	Code Included in Restrictive^a^ Scenario?
*Non-FQHCs*		
CPT 99484	Care Management Services for Behavioral Health Conditions (General BHI)	No
CPT 99492	Initial CoCM (first month)	Yes
CPT 99493	Follow Up CoCM (subsequent month)	Yes
CPT 99494	Add-on CoCM, any month (initial and subsequent)	Yes
HCPCS G2214	CoCM Management	No
*FQHCs*		
HCPCS G0511	Care Management Services for Behavioral Health Conditions (General BHI)	Yes
HCPCS G0512	Psychiatric CoCM (any month)	Yes

BHI = Behavioral Health Integration. CoCM = Collaborative Care Model. CPT = Current Procedural Terminology. FQHC = Federally Qualified Health Center. HCPCS = Healthcare Common Procedure Coding System.

^a^We assumed all codes listed in the table were billable in the expansive scenario.

Sources: (Centers for Medicare & Medicaid Services, 2023a, 2023b).

Each CoCM or BHI code had service and time requirements that must be documented to bill the code. We operationalized these requirements for each calendar month of care for each patient using available registry data, as summarized in Tables 2 and 3. Furthermore, before starting and billing for CoCM services, patients must verbally consent to the model of care (Centers for Medicare & Medicaid Services, 2023a). Consent to participate in the trial included agreeing to potentially receive CoCM, but care managers also obtained verbal consent for CoCM during initial patient engagement (a typical consent process in practice settings).

**Table 2:** Operationalization of service requirements for billing codes used with the collaborative care model

Billing Code	Service Requirement^a^	Operationalization for Calendar Month
All CoCM codes (99492, 99493, 99494, G2214, G0512)	• Participation in weekly caseload consultation with the psychiatric consultant (with modifications of the plan, if recommended)	• Patient had BHC consultation
	• Provision of brief interventions using evidence-based techniques such as behavioral activation, motivational interviewing, and other focused treatment strategies	• Patient had care manager encounter
	• Tracking patient follow-up and progress using the registry, with appropriate documentation	• Patient had entry in registry
Initial month for CoCM codes (99492, 99494, G0512^b^)	• Outreach to patient and engagement in treatment that is directed by the treating physician or other qualified health care professional	• Patient had care manager encounter
	• Initial assessment of the patient, including administration of validated rating scales	• Patient had responses to any MBC measure included in the registry
	• Development of an individualized treatment plan	• Patient had BHC consultation (because treatment plans were not captured in the registry)
Follow-up months for CoCM codes (99493, 99494, G0512^b^)	• Monitoring patient outcomes using validated rating scales	• Patient had responses to any MBC measure included in the registry
	• Ongoing collaboration with and coordination of the patient’s mental health care with the treating physician or other qualified health care professional and any other treating mental health providers	• Patient had BHC consultation (because other between-provider communication was not captured in the registry)
	• Relapse prevention planning with patients as they achieve remission of symptoms or other treatment goals and are prepared for discharge from active treatment	• Patient had care manager encounter and BHC consultation (because relapse prevention plans were not captured in the registry)
General BHI codes (99484, G0511)	• Initial assessment or follow-up monitoring, including using applicable validated rating scales	• Patient has responses to any MBC measure included in the registry
	• Behavioral health care planning about behavioral or psychiatric health problems, including revision for patients not progressing or whose status changes	• Patient has care manager encounter
	• Facilitating and coordinating treatment such as psychotherapy, pharmacotherapy, counseling, or psychiatric consultation	• Patient has care manager encounter
	• Continuity of care with an appointed member of the care team	• Assumed always met because all patients were assigned to a care manager

BHC = behavioral health consultant (i.e., term we used for our team’s psychiatric consultants, who were board-certified in addiction medicine or addiction psychiatry; the billing codes do not specify any requirements that psychiatric consultants use that title or hold those board certifications). BHI = Behavioral Health Integration. CoCM = Collaborative Care Model. MBC = measurement-based care.

^a^Required component language is adapted from: (AIMS Center, 2023d; Centers for Medicare & Medicaid Services, 2023a).

^b^G0512 can be billed for initial or subsequent months but has different minimum time requirements depending on the month.

We calculated eligible and ineligible minutes overall for the sample, as well as by FQHC vs. non-FQHC setting, and for the expansive vs. restrictive billing scenarios. We also calculated what percentage of minutes were ineligible due to which criteria (e.g., did not meet specific service or time requirements). Finally, we examined the proportion of ineligible minutes that occurred prior to patient engagement in CoCM, defined as the first month the patient met all the service requirements for CoCM (described next).

**Table 3:** Time requirements for billing codes used with the collaborative care model

Billing Code	Time Restriction for Calendar Month	Billing Frequency
*Non-FQHCs*		
99484 (General BHI)	• 20 minutes	• Once per month • Any month in which CoCM is not billed
99492 (CoCM initial)	• 36–70 minutes^a^	• Once in the first CoCM month
99493 (CoCM follow-up)	• 31–60 minutes^a^	• Once per month • Subsequent CoCM months after initial
99494 (add-on CoCM)	• Additional 16–30 minutes^a^ (beyond those billed to 99492/99493)	• Up to twice per month • Any month
G2214 (CoCM management)	• 16–30 minutes^a^	• Once per month; only if 99492/99493 cannot be billed • Any month
*FQHCs*		
G0511 (General BHI)	• 20 minutes	• Once per month • Any month in which G0512 cannot billed
G0512 (CoCM)	• 70 minutes for first month • 60 minutes for subsequent months	• Once per month • Any month in which G0511 is not billed

^a^CPT half-plus-one time rule applies, which allows for billing of services starting at 50% plus one minute of the maximum time allowed

Sources: AIMS Center (2023d); Centers for Medicare & Medicaid Services (2023a, 2023b)

#### Service Requirements

Several components were required across all CoCM codes (99492–99494, G2214, and G0512) (AIMS Center, 2023d; Centers for Medicare & Medicaid Services, 2023a). As shown in Table 2, we operationalized the caseload consultation requirement as documentation of one BHC consultation during the month. Although billing guidelines and the trial both required weekly BHC caseload consultation, CMS has confirmed that each individual patient may not require discussion during every weekly caseload review (AIMS Center, 2023c). Thus, we took at least one documented consultation in a month as indicating the care manager regularly met with the BHC and discussed the individual patient as needed. We operationalized the brief intervention requirement as the patient having at least one care manager encounter in the month, given these encounters involved evidence-based care interventions (e.g., motivational interviewing) as needed. For the third component, tracking patient follow-up and progress, we required at least one registry entry for the patient in the month. A fourth required component for CoCM codes was use of MBC, though this requirement varied somewhat between initial and subsequent months (shown separately in Table 2). We operationalized these requirements as needing documented evidence of responses to at least one of the registry’s MBC measures. We defined complete responses as ≥50% items answered or using guidance from the measure’s source literature, if available.

Our operationalization of the primary CoCM service requirements as documented care manager encounter, BHC consultation, and MBC administration aligns with Belsher et al.’s operational definition of fidelity to CoCM (Belsher et al., 2018). We used these same activities to operationalize code-specific requirements (for e.g., engagement, treatment planning, team-based communication), as shown in Table 2. More detailed documentation of those activities was unavailable to us because it was done in the electronic medical record, but all the activities were directly supported by BHC consultation and care management processes.

The general BHI codes (99484, G0511) did not require all CoCM components. The codes still required care management and MBC, but treatment coordination did not have to involve BHC consultation or a registry (Centers for Medicare & Medicaid Services, 2023a). We also assumed the continuity of care requirement for general BHI was fulfilled by the designated care manager for all patients.

#### Time Requirements

Each billing code had restrictions on how often it can be billed and how much care manager time can be reimbursed per month, as summarized in Table 3. CoCM codes for non-FQHCs followed a “half-plus-one” CPT time rule in which they could be billed once services reached one minute over half of the maximum billable time for a given month (American Medical Association, 2021). For codes not following this rule, a set amount of time is covered instead of a range.

If all three CoCM service requirements were met, we first attempted to apply CoCM codes (99492, 99493, G0512) depending on the setting and, if relevant, initial versus follow-up month. If the month was under these codes’ minimum billing times, we attempted to apply G2214 for non-FQHCs or G0511 for FQHCs; if those time requirements also were not met, then the minutes were unbillable. Minutes exceeding the maximum for G2214/G0511 but below the minimum for 99492/G0512 were also considered unbillable. If a month exceeded the maximum allowable time for 99492 or 99493 for non-FQHCs, we then attempted to apply 99494; any minutes exceeding the maximum for 99492/99493 and two instances of 99494 were unbillable. Any minutes at FQHCs that exceeded the maximum for G0512 were also unbillable.

If only the care manager encounter and MBC were present, we could not apply CoCM codes but attempted to apply General BHI codes instead (99484/G0511). Additionally, for any months that had the CoCM-required services but did not meet the minimum minutes for billing an initial month of CoCM code, we attempted to bill the general BHI code for that month and attempted to bill the initial month CoCM code the following month.

#### Reimbursement Potential

Finally, we calculated estimated reimbursement rates per hour of care manager work. To do so, we first identified the 2024 non-facility payment amounts for each code in the CMS fee schedule (Centers for Medicare & Medicaid Services, 2024) as summarized in Table 4. Then, for each month and patient, we calculated the reimbursement possible based on which billing code(s) were determined eligible for billing. We then summed all reimbursements across patients and months, and we divided this dollar amount by the sum of all documented time spent on care manager activities. This produced totals for potential reimbursement and hours of CoCM delivery, plus an hourly rate for potential reimbursement; again, this was done overall, for FQHCs versus non-FQHCs, and for expansive versus restrictive billing conditions.

**Table 4:** Medicare reimbursement rates for billing codes used with the collaborative care model

Billing Code	Reimbursement Rate^a^
*Non-FQHCs*	
99484	$54.92
99492	$153.12
99493	$139.81
99494	$59.25
G2214	$57.25
*FQHCs* ^b^	
G0511	$83.88
G0512	$146.57

FQHC = Federally Qualified Health Center

^a^Reimbursements are per-patient, per billing instance

^b^Calculated from relevant non-FQHC rates per CMS guidance

Source: Centers for Medicare & Medicaid Services (2024)

### Sensitivity Analyses

To evaluate the impact of uncertainty in key assumptions made for the analyses, we also conducted three sensitivity analyses, by substituting minimum and/or maximum plausible values into the analysis: (1) We varied reimbursement rates to align with minimum and maximum Medicaid rates rather than Medicare. We researched state regulations and found that among states whose Medicaid programs reimbursed for CoCM, most reimbursed at rates below Medicare (Meadows Mental Health Policy Institute, 2025). Based on available data (Arkansas Behavioral Health Integration Network, 2023), the lowest 2024 CoCM reimbursement rates among state Medicaid programs were those of Pennsylvania (Pennsylvania Department of Human Services, 2025); the highest rates were those of Montana (Montana Department of Public Health and Human Services, 2024), one of the few states that reimbursed above Medicare. (2) We calculated total CoCM minutes assuming each contact attempt took 10 min instead of five; according to care managers and their supervisors, 10 min was the maximum plausible length. (3) We tested the potential impact of limited billing capacity by assuming it was plausible that up to 40% (i.e., slightly less than half) of months with minutes eligible to be billed would not actually be billed by primary care practices actively using the CoCM codes.

## Results

### Participants

Among the 381 adult patients with opioid use disorder who received CoCM, 55% were female, 69% were Hispanic, and 41% completed some college or more. They had an average age of 40.1 years (SD = 12.25), 92% were from New Mexico (vs. California), and 50% were from the FQHC system. Most patients (58%) had both co-occurring mental disorders, compared with only post-traumatic stress disorder (22%) or only depression (20%).

### Total CoCM Minutes

Care managers documented 90,996 min of CoCM delivery in the project’s registry. Of these minutes, 11.2% were associated with initial encounters between the patient and the care manager; 50.4% with follow-up care manager encounters; 12.3% with BHC consultations; and 26.1% with contact attempts (assuming 5 min each). Table 5 details the breakdown of total minutes by encounter type.

**Table 5:** Summary of CoCM minutes from registry entries, by encounter type

Encounter Type	Total Minutes	Count of Registry Entries	Minutes per Entry Mean (SD)
Initial care manager encounter	10,216 (11.2%)	314	33 (17)
Follow-up care manager encounter	45,839 (50.4%)	1,925	24 (14)
Contact attempt	23,735 (26.1%)	4,747	5 (0)^a^
BHC consultation	11,206 (12.3%)	1,582	7 (5)

BHC = Behavioral Health Consultant (i.e., term we used for our team’s psychiatric consultants, who were board-certified in addiction medicine or addiction psychiatry; the billing codes do not specify any requirements that psychiatric consultants use that title or hold those board certifications). CoCM = Collaborative Care Model.

^a^Length of contact attempts was not documented, so we assumed five minutes per attempt.

### Reimbursement Potential Based on Eligible Minutes

**Fig. 1:**
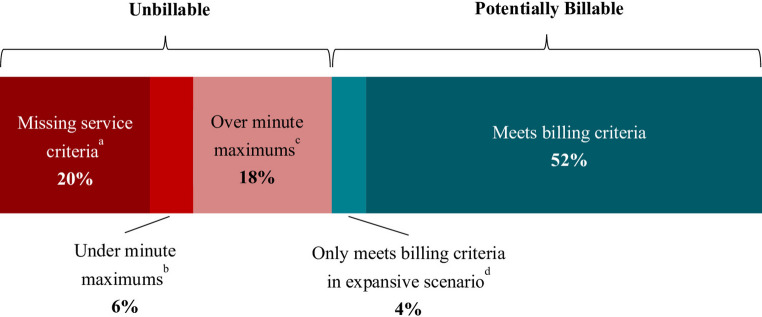
Billing eligibility of all CoCM and BHI minutes with reasons for ineligibility. *Note.* BHI = Behavioral Health Integration. CoCM = Collaborative Care Model. ^a^Missing CoCM and General BHI billing code service requirements (see Table 2 for details). ^b^Meets service requirements but total minutes for month fall under minimum allowable minutes for CoCM or General BHI billing codes (see Table 3 for details).^c^Meets service requirements but total minutes for month exceed maximum allowable minutes under all possible combinations of CoCM and BHI billing codes (see Table 3 for details). ^d^Meets service and time requirements, but only billable under billing codes included in the expansive billing condition; unbillable in the restrictive condition (see Table 1 for details)

Table 6 shows the calculations for total time and potential reimbursement in each scenario and facility type. Overall, of the 90,996 min, 56% (51,379) were eligible for reimbursement under the expansive billing scenario versus 52% (47,299) under the restrictive billing scenario. These eligible minutes translated to an average reimbursement per hour of $91.61 and $84.53 respectively. Figure 1 characterizes the breakdown of these minutes, including whether minutes were ineligible due to missing service requirements or time requirements (and for the latter, if over maximum vs. under minimum). Table 7 further shows how the eligible minutes were distributed across billing codes.

**Table 6:** Summary of CoCM minutes eligible for billing codes and potential reimbursement, by scenario and setting

Variable	Analytic Scenario^a^ and Setting(s)					
	Expansive			Restrictive		
	Combined	Non-FQHCs	FQHCs	Combined	Non-FQHCs	FQHCs
Total minutes	90,996	53,091	37,905	90,996	53,091	37,905
Total hours	1,517	885	632	1,517	885	632
Minutes eligible to bill	51,379	32,529	18,850	47,299	28,449	18,850
Percent of total minutes eligible to bill	56%	61%	50%	52%	54%	50%
Potential reimbursement from eligible CoCM time	$138,943.19	$80,930.70	$58,012.49	$128,202.50	$70,190.01	$58,012.49
Potential reimbursement per hour of CoCM^b^	$91.61	$91.46	$91.83	$84.53	$79.32	$91.83

CoCM = Collaborative Care Model. FQHC = Federally Qualified Health Center.

^a^See Table 1 for details of which billing codes were included in the expansive vs. restrictive scenarios; note that the FQHC codes were the same in both scenarios.

^b^Potential reimbursement divided by total hours.

For non-FQHCs, 61% of minutes were billable under the expansive scenario and 54% of minutes were billable under the restrictive scenario; the average reimbursements per hour were $91.46 and $79.32 respectively. For FQHCs, 50% of minutes were billable (identical in both scenarios), with a corresponding average reimbursement per hour of $91.83.

**Table 7:** Billable CoCM and BHI minutes by billing code

Billing Code	Minutes Eligible to Bill	Percentage^a^
*Non-FQHCs*		
99492	7,763	24%
99493	14,526	45%
99494, billed once	4,119	13%
99494, billed second time	2,041	6%
99484	3,140	10%
G2214	940	3%
*FQHCs*		
G0511	8,000	42%
G0512	10,850	58%

CoCM = Collaborative Care Model. FQHC = Federally Qualified Health Center.

^a^ Percentage out of all billable minutes for this type of practice (Non-FQHC or FQHC). Percentages may not sum to 100 due to rounding.

#### Minutes Ineligible for Reimbursement

For the full sample, 44% of minutes were ineligible for reimbursement, even under the expansive scenario (see Fig. 1). Minutes were considered ineligible if they: (1) did not meet one or more service requirements or (2) fell outside the time requirements.

**Fig 2.**
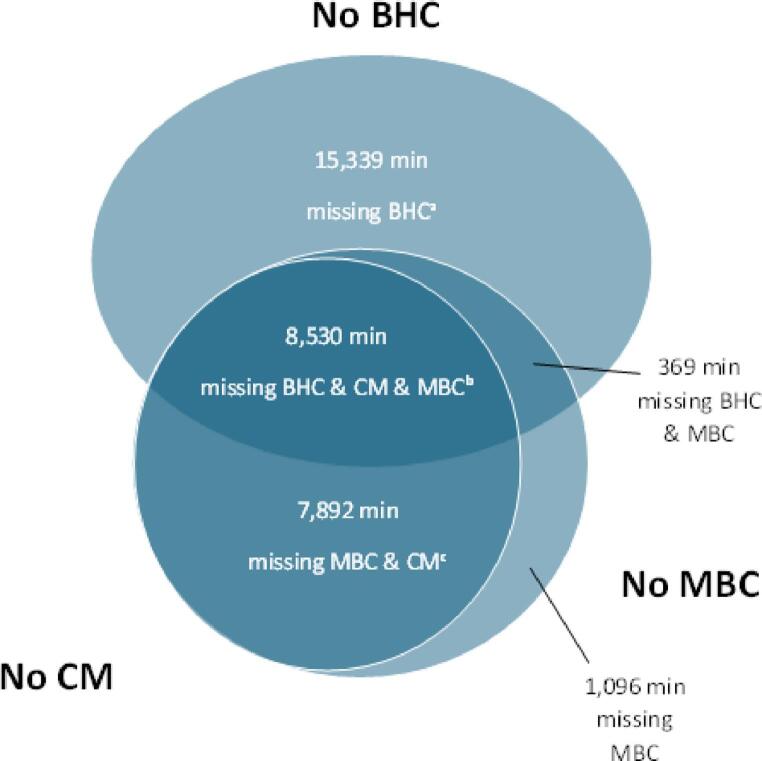
Missing service requirements that led to CoCM billing ineligbility*Note*. CM = Care Manager encounter with patient; BHC = Behavioral Health Consultant caseload consultation (BHC was the term we used for our team’s psychiatric consultants, who were board-certified in addiction medicine or addiction psychiatry; the billing codes do not specify any requirements that psychiatric consultants use that title or hold those board certifications); MBC = Measurement Based Care documented in registry. See Table 2 for details of service requirements. ^a^The 8,530 minutes occurring in months that are missing all three service requirements were minutes the Care Manager spent on contact attempts to reach patients. ^b^Months missing only a BHC consultation do not meet service criteria for CoCM codes but do meet service criteria for general BHI codes. ^c^MBC must occur during a CM encounter, so all minutes that meet the MBC requirement also meet the CM encounter requirement

Figure 2 shows a detailed breakdown of minutes ineligible for CoCM reimbursement due to missing one or more of the three CoCM service requirements. Overall, 8,530 of these minutes lacked all three service requirements. The majority of ineligible minutes were missing a BHC consultation, but when this was the only service requirement lacking (15,339 min), the General BHI codes offered a source of potential reimbursement. Still, 17,887 min (20% of all minutes) were ineligible for reimbursement of any sort because they did not meet one or more of the General BHI service requirements (see Table 2).

Moreover, 21,730 minutes (24% of all minutes) were ineligible for reimbursement because they did not meet time requirements for billing codes (see Table 3). Of those ineligible minutes, over three-quarters (16,527 min, 18% of total) were time spent *above* maximum allowable time, compared to those *below* minimum allowable time. This translated to a ratio of 3.2 minutes spent over billing code maximums for every one minute spent under the minimums.

Finally, among the 39,617 total minutes that were unbillable to any CoCM or BHI code, 13,409 min (34% of all unbillable minutes) occurred prior to the first month in which all three CoCM service criteria were met (i.e., care manager encounter, MBC, BHC consultation). This unbillable time spent prior to patient engagement in CoCM comprised 15% of all minutes.

### Sensitivity Analyses

Varying our assumptions about billing generally reduced reimbursement potential and the percentage of minutes that were reimbursable. The results of all sensitivity tests are summarized in the Appendix Table. Reimbursement varied most widely when testing the minimum and maximum plausible state Medicaid rates (sensitivity test 1); it was as low as $38.43 per hour with minimum Medicaid rates (Pennsylvania) among non-FQHCs and as high as $115.71 per hour with maximum Medicaid rates (Montana) among FQHCs. Reimbursement potential was also lower than the main findings when we assumed 10-minute contact attempts (sensitivity test two) or that a portion of eligible months were not billed (sensitivity test three). The Appendix figure illustrates the comparison between our main findings, i.e. “ideal” conditions, with findings from test three assuming that 40% of months with eligible minutes not being billed in practice.

## Discussion

This study examined the reimbursement potential of CoCM billing codes with patients who have opioid use disorder co-occurring with depression and/or post-traumatic stress disorder. We found that a notable proportion of care manager time spent delivering CoCM was not billable (44% under expansive billing conditions, up to 48% under restrictive conditions), resulting in potential reimbursement (with 2024 Medicare rates) of $79.32 to $91.83 per hour of care manager effort across over 1,500 hours of CoCM activities. Despite FQHCs using billing codes with higher reimbursement rates, non-FQHCs were able to achieve comparable potential reimbursement per hour of CoCM – but only in the expansive billing scenario. Sensitivity analyses that varied key assumptions showed that in most cases where our assumptions do not hold, reimbursement potential would be notably lower. These findings can help health system and policy decision-makers understand how available financing aligns with CoCM delivery to patients with co-occurring mental and substance use disorders, a high-need and clinically complex population. Strengths of our study include a large, diverse sample of clinics and patients; standardized intervention delivery and documentation processes; and careful operationalization of CoCM billing codes and rules across a range of plausible scenarios.

Notably, we found two mismatches between (a) necessary intervention activities for patients with co-occurring mental and substance use disorders and (b) allowable expenses under the CoCM billing codes; these mismatches limit reimbursement potential. First, considerable outreach is needed for these patients, who may be less engaged in primary care due to their complex behavioral health and social needs (Huynh et al., 2016; Snell-Rood et al., 2021). The billing codes assume that patients are already engaged and initiate CoCM with a medical provider (Centers for Medicare & Medicaid Services, 2023a), but our care managers had to provide extensive outreach services that could not be billed fully until the patient engaged (i.e., met CoCM service requirements; 15% of all minutes). Second, a higher proportion of minutes from our sample exceeded the maximum time allowed by billing codes (18%) compared to a study of CoCM with mental disorders (i.e., 1–13%; Chang et al., 2023). Once engaged, time spent on care coordination exceeded levels supported by the codes, consistent with treating those with complex needs (Leamon et al., 2024; Snell-Rood et al., 2021; Winiker et al., 2023). Modifications to the rules governing the codes to minimize unbillable labor required to deliver CoCM with high-need populations (e.g., reimbursement for initial engagement and management of clinical complexity) could help support CoCM delivery.

Understanding the current reimbursement potential for CoCM is complex and warrants a formal cost analysis, which was outside of the current study’s scope. That said, the 2023 median national wage estimates for relevant occupational categories (i.e., community and social service) were ~$20–30 per hour, plus the 32% median fringe benefit rate (U.S. Bureau of Labor Statistics, 2023, 2024). Health system overhead rates must also be considered (e.g., at least the 10% federal *de minimis* rate; per 2 CFR 200.68), including billing systems that make reimbursement possible. This already represents a median cost of approximately $29–44 per hour of care management. Furthermore, there can be CoCM-specific expenses for the patient registry, care manager supervisor (when care managers are not licensed, independent practitioners, as in our study), behavioral health consultant, and/or measurement-based care (Advancing Integrated Mental Health Solutions [AIMS] Center, 2014). Total costs appear most feasible to cover at Medicare rates under expansive CoCM billing conditions or in FQHCs, but other scenarios that we tested (e.g., restrictive billing options, lower reimbursement rates) apply in many non-FQHC contexts (Raney, 2020) or when billing Medicaid. Moreover, higher salaries are likely appropriate to recruit and retain care managers who have the skills needed to support patients with both mental and substance use disorders. Finally, ongoing analyses will be needed to understand how CoCM reimbursement potential shifts in response to changing policy conditions; for example, CMS (2026) has now introduced new Medicare billing rules for FQHCs that mirror non-FQHC rules.

Numerous factors beyond fee-for-service reimbursement influence the feasibility and long-term sustainability of CoCM delivery. Critically, additional supports are needed to enable scale-up of evidence-based practices like CoCM among primary care practices and providers that are often overextended already, including technical support for using these complex codes (Carlo et al., 2019, 2020). Introduction of the BHI codes resulted in < 0.1% of eligible beneficiaries receiving CoCM services billed under those codes, per studies examining Medicare (Cross et al., 2020) and a state Medicaid program (Copeland et al., 2022; Davenport et al., 2025). Provider hesitance to use the CoCM codes persists (Little et al., 2022) and may be especially challenging in states that have been late to adopt the CoCM codes for Medicaid (Raney, 2020). Primary care clinics might also use additional funding sources to implement and sustain CoCM, such as grants and contracts as well as value-based or other alternative payment models (Dopp et al., 2020). Cross et al. specifically recommended capitated monthly per-patient payments, tied to patient diagnosis and use of evidence-based practices, for CoCM. Clinic leaders could benefit from strategic planning tools such as the Fiscal Mapping Process (Dopp et al., 2023, 2024) that guide coordination of multiple funding sources across all aspects of CoCM. Moreover, primary care clinics may need to invest additional resources into thoughtfully modifying CoCM to promote implementation and sustainment (Salloum et al., 2022); for example, the clinics in this study have all considered expanding their CoCM services beyond co-occurring disorders to broadly included patients with mental and/or substance use disorders.

Our findings should be interpreted in light of six study limitations. First, our data came from a CoCM patient registry used by community health workers in a research trial, and caution is warranted when generalizing these estimates to predict potential reimbursement in clinical settings and/or with care managers from other backgrounds. Second, our findings likely generalize to only primary care clinics with robust systems (e.g., registry, integrated medical record systems) for tracking CoCM activities to enable billing; and, even for well-resourced clinics, the documentation burden and associated audit risk (i.e., if documentation is not performed correctly) may disincentivize use of CoCM billing codes. Third, though we modeled reimbursement potential for Medicare versus Medicaid through sensitivity testing, we lacked access to sufficient information to perform a comparison to reimbursement potential for commercial payers as well. Fourth, policymakers and clinic leaders may not directly use our estimates in decision-making because the adequacy of CoCM reimbursement depends on (a) the cost structure of CoCM services, (b) the billing codes and reimbursement rates offered in their policy context, and (c) the payor mix for eligible patients. Given this complexity, decision-makers would benefit from practical guidance such as a simulation model that calculates reimbursement potential based on user-specified values. Fifth, we required monthly BHC notes to consider minutes billable, which may have produced conservative estimates of reimbursement potential; we found limited guidance on how that requirement can be documented, and in practice, CoCM teams may use methods less burdensome than patient-specific notes (e.g., noting the patient was considered for consultation in a summary of monthly billable activities).

Finally, formal economic evaluations of CoCM for co-occurring mental and substance use disorders are needed to supplement the well-established cost-effectiveness of the model for mental disorders (Palinkas et al., 2011). Full accounting of CoCM costs requires incorporating activities beyond care manager time, including both costs and total revenue across CoCM billing codes and other sources, such as billing for medical and mental health interventions (e.g., Chang et al., 2023). Moreover, economic evaluations can account for broader societal perspectives – e.g., effects of treated versus untreated disease on medical spending (Wolk et al., 2023), quality of life, functioning, and mortality – to inform when and how investing in CoCM is worthwhile.

In conclusion, continued attention is needed to understand reimbursement for CoCM with patients who have co-occurring mental and substance use disorders. By modifying the CoCM billing codes to begin covering unbillable activities (e.g., initial engagement, management of clinical complexity), in coordination with comprehensive implementation supports, policymakers and health system leaders can maximize the feasibility and sustainability of an evidence-based practice designed for treatment of high-need patients in primary care.

## Supplementary Information

Below is the link to the electronic supplementary material.

Supplementary Material 1

## Data Availability

Data from this research project will be made available through the NIMH Data Archive upon project completion per the terms of grant U01MH121954. Interested parties can contact the investigators with inquiries about which data will be shared, and alternative options for data sharing in cases where data of interest will not be included in the Data Archive.
