# Supplementary material for: Reimbursement Potential of Collaborative Care Model (CoCM) Billing Codes for Opioid Use Disorder Co-Occurring with Mental Disorders: Descriptive Estimates from a Pragmatic Trial
Source: Adm Policy Ment Health. Author manuscript; Available in PMC 2026 Jun 2. (PMC13225723; doi:10.1007/s10488-026-01503-z)
Supplement: Supplementary file 1 [file NIHMS2164425-supplement-Appendix.docx]

Appendix

Table 8

Sensitivity Analyses Findings: Minimum and Maximum Plausible Reimbursement Potential Per Hour, by Analysis and Setting

	Reimbursement per hour, by analysis				
	Main analysis	Sensitivity test one: Varying reimbursement rates to align with Medicaid		Sensitivity test two: 10 minute contact attempts	Sensitivity test three: 40% of eligible months are not billed
Setting		Minimum: Pennsylvania[a]	Maximum: Montana[b]	Minimum	Minimum
Overall	\$84.53 - \$91.61	\$39.63 - \$43.04	\$102.79 - \$109.88	\$71.30 - \$76.70	\$49.90 - \$54.22
Non-FQHC	\$79.32 - \$91.46	\$38.43 - \$44.27	\$93.57 - \$105.71	\$66.25 - \$75.24	\$45.87 - \$53.26
FQHC	\$91.83	\$41.32	\$115.71	\$78.92	\$55.55

Note. FQHC = Federally Qualified Health Center.
Ranges within a cell represent the full range of reimbursement rates per hour from the restrictive and expansive billing scenarios, for that analysis and settings. For sensitivity tests two and three, the maximum plausible reimbursement value is equivalent to the main analysis findings.
[a]Taken from Pennsylvania Department of Human Services (2025)
[b]Taken from Montana Department of Public Health and Human Services (2024)

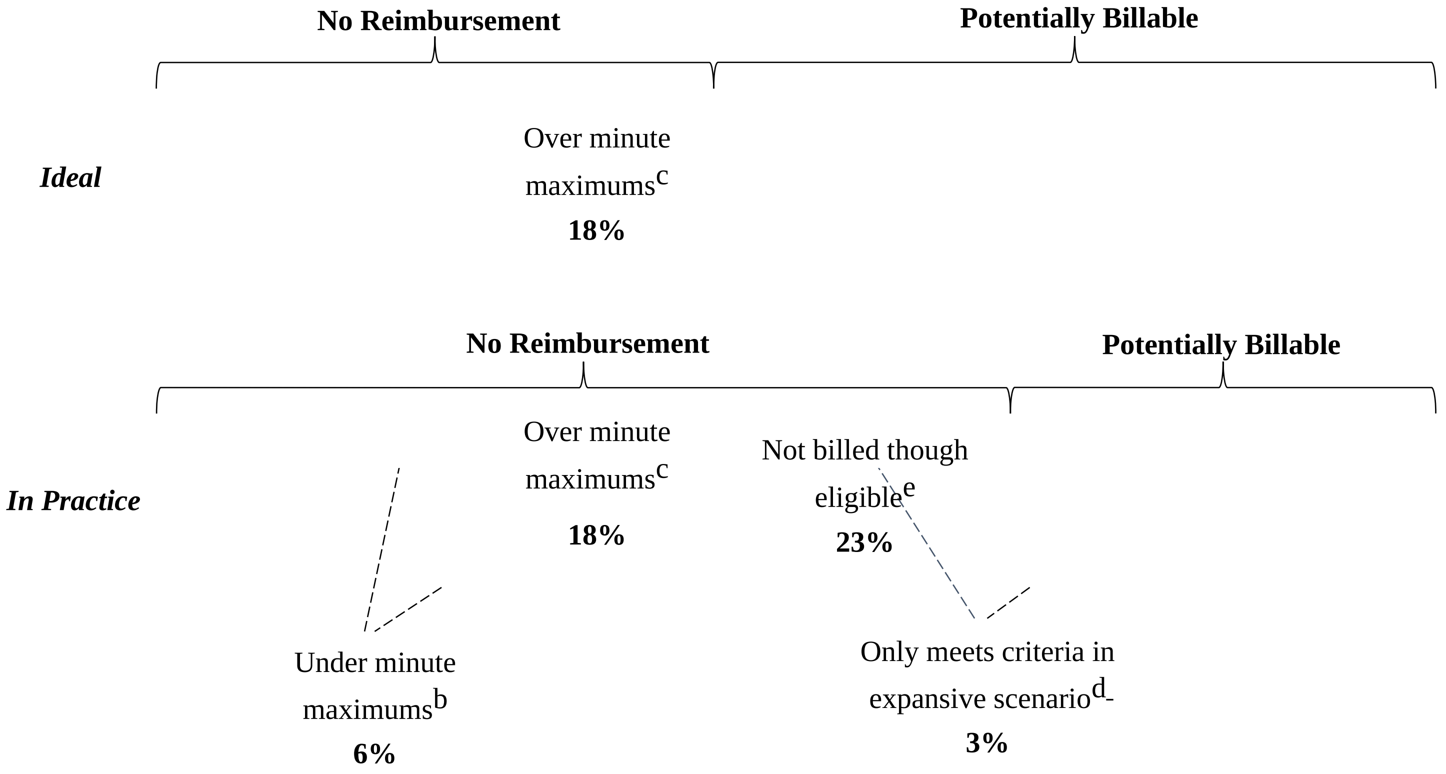

Figure 3. *Reimbursement Potential if 40% of Eligible Months Are Not Billed in Practice*

Note. BHI = Behavioral Health Integration. CoCM = Collaborative Care Model.
[a]Missing CoCM and General BHI billing code service requirements (see Table 2 for details).
[b]Meets service requirements but total minutes for month fall under minimum allowable minutes for CoCM or General BHI billing codes (see Table 3 for details).
[c]Meets service requirements but total minutes for month exceed maximum allowable minutes under all possible combinations of CoCM and General BHI billing codes (see Table 3 for details).
[d]Meets service and time requirements, but only billable under billing codes included in the expansive billing condition; unbillable in the restrictive condition (see Table 1 for details).
[e]Meets service and time requirements but is not billed due to other reasons.
